# Long-term follow-up of thumb reconstruction with a heterotopic replanted finger: case report and literature review

**DOI:** 10.3389/fbioe.2024.1465108

**Published:** 2024-11-08

**Authors:** Zhihu Ma, Xingsheng Zhang, Gang Wang, Lei Zhu, Yantao Pei, Yuliang Sun, Ben Liu

**Affiliations:** Department of Orthopedics, Department of Hand Surgery, Qilu Hospital of Shandong University, Jinan, China

**Keywords:** thumb reconstruction, heterotopic replantation, microsurgery, hand function, severed fingers

## Abstract

**Objectives:**

This work aimed to study the long-term outcome and function of a heterotopic replanted finger.

**Methods:**

Retrospective analysis of two cases of successful finger reconstruction after finger heterotopic replantation. One case was a severed thumb, and the other case was a severed finger. The average follow-up time was 10 years. The clinical outcome and function of the heterotopic replanted finger, including range of motion, grip strength, and sensory recovery, were analyzed,. A comparative analysis was conducted with patients who underwent thumb replantation in the same period.

**Results:**

The two heterotopically replanted fingers survived. Five months after the heterotopic replantation surgery, a new thumb was reconstructed using the heterotopically replanted thumb and the free tarsometatarsal joint from the foot. In the other case, the finger was reconstructed 1 year later with a free anterolateral thigh flap and the heterotopically replanted finger. The movement of the thumb, the pinching of the fingers, the clenching of the fist, and the feeling recovery were all good. The patient was capable of independently performing daily life and work activities such as eating, dressing, writing, and so on. There was no significant difference in hand function between the patients who received *in situ* finger replantation and heterotopic replantation during the same period.

**Conclusion:**

For severed fingers that cannot be replanted *in situ* in an emergency situation, heterotopic replantation and secondary reconstruction may be a feasible and practical surgical method.

## 1 Introduction

Finger amputation is a common and serious disease type in hand trauma, and finger replantation may be the best method to save the severed fingers. Studies have shown that the survival rate of replantation of severed fingers can reach about 90%, and the recovery of hand function is between 50% and 90% (Erçin et al., 2022; Kaneshiro et al., 2020; Chang et al., 2015). Patients with complex severed fingers due to more severe soft tissue injuries have a low reimplantation survival rate. Moreover, their postoperative care is more expensive. Based on the patient’s requirements and the development of microsurgical techniques, replantation will be attempted for most severed fingers, including shortened replantation, flap transplantation with finger replantation, or thumb reconstruction (Wang and Sun, 2014), and even cryopreservation and replanting (Wang et al., 2020; Wang Z. et al., 2019).

Godina’s team first described a method of saving a limb by temporarily implanting the undamaged distal limb in an ectopic position in 1986. The literature review indicates that heterotopic replantation has been used in clinical practice and achieved good results. It is a reliable and valuable innovative technology for limb rescue and reconstruction (Buda et al., 2018; Cho and Higgins, 2019). It is recommended for patients with devastating segmental or soft tissue injuries who face difficulty in early assessment of the necrotic area of the injured limb for immediate revascularization, multiple organ injury, hemodynamic instability for immediate prolonged replantation, or heavy contamination or tissue loss at the stump of an amputation (Cho and Higgins, 2019; Cavadas et al., 2011; Wang et al., 2005). For patients with severe finger injuries, temporary heterotopic replantation can improve the survival rate of replantation and offer good functional recovery (Del, 2019).

In this study, due to the relatively serious injury, after a comprehensive assessment, heterotopic finger replantation was selected by the patients. One thumb was replanted on the dorsal side of the healthy hand for approximately 5 months, and one small finger was replanted on the healthy hand for approximately 1.5 years. When the patients’ conditions improved, the heterotopic replanted fingers were successfully replanted back to their original position. After long-term follow-up and retrospective study, the appearance and function of the replanted fingers were evaluated.

## 2 Case description

### 2.1 Patients

**FIGURE 1:**
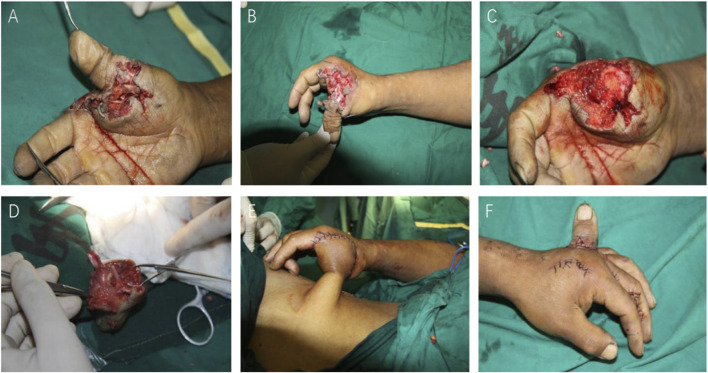
**(A, B)** Complete finger amputation of the thumb, with an irregular wound edge and wound pollution. The metacarpophalangeal joint was completely damaged. **(C, D)** Debridement of the wound and the thumb. **(E)** The thumb stump wound was repaired by a pedicled inguinal flap. **(F)** The severed thumb was replanted on the dorsal side of the left hand.

From March 2011 to October 2014, three patients received temporary heterotopic finger replantations, and two of the patients elected transplantation of their heterotopic replanted fingers back to their original site in the second stage; the other patient chose to terminate the replantation. The first patient was a 38-year-old man whose right thumb was completely severed. The proximal soft tissue and metacarpophalangeal joint were completely damaged, which rendered him unfit for emergency replantation. (Figures 1A, B). The severed right thumb was heterotopic replanted on the back of the left hand. The finger artery was anastomosed to the radial digital artery of the middle finger, and the finger vein was anastomosed to the dorsal vein of the palm. The thumb stump wound was repaired by a pedicled groin flap (Figures 1C–F). The second patient was a 22-year-old male soldier with multiple composite tissue injuries and debridement injuries to the right hand, with the index and middle fingers completely severed. The severed fingers were heterotopically replanted on the back of the left hand. Both patients were denied any underlying disease or family history of other diseases as contraindications to replantation. Informed consent was given prior to surgery. In accordance with the Declaration of Helsinki, our study was approved by Shandong University Qilu Hospital(QLCR20230669), and informed consent was obtained from the patients.

### 2.2 Surgical methods

**FIGURE 2:**
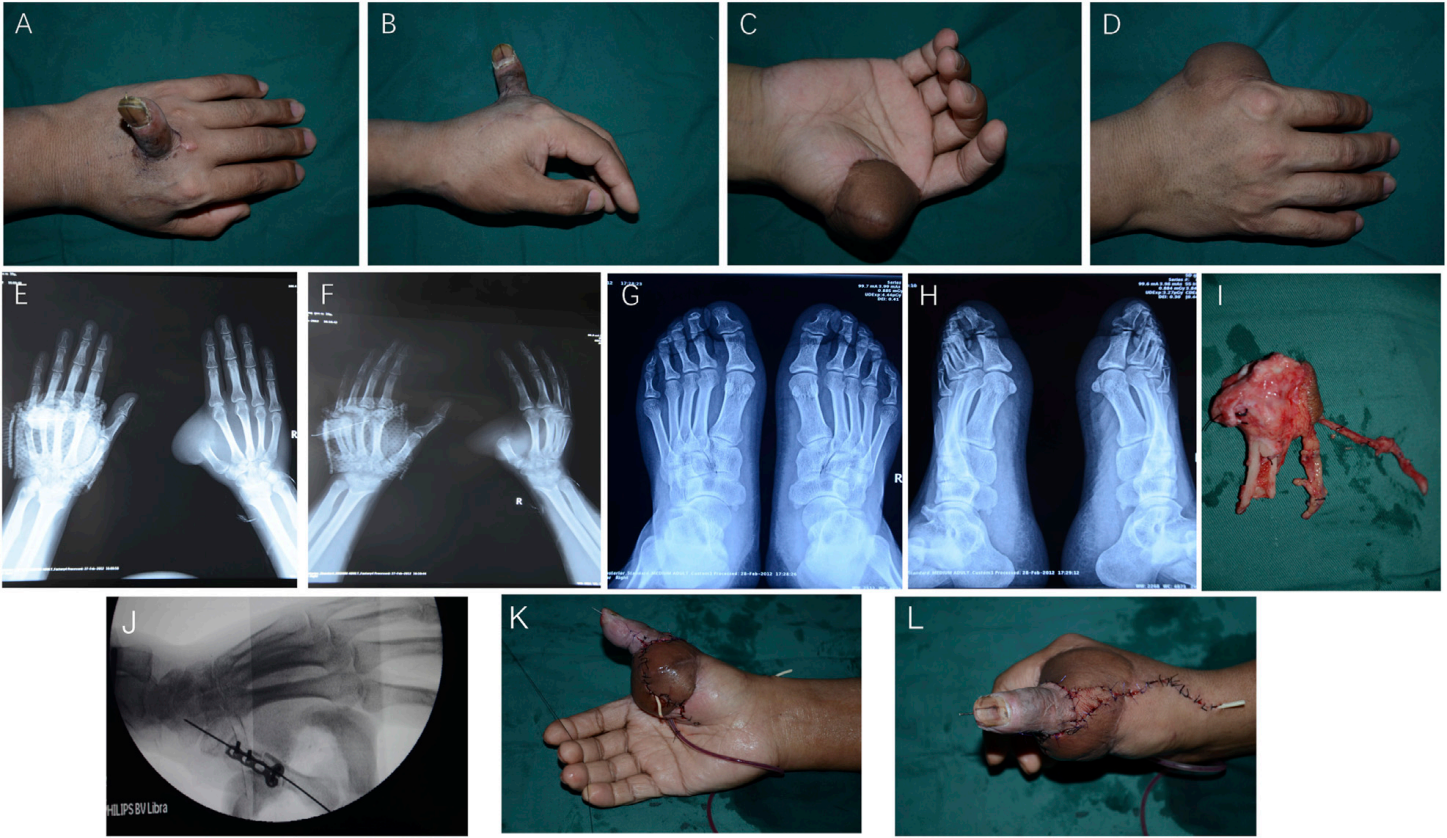
Thumb heterotopic replantation 5 months after the injury. **(A, B)** The wound was repaired by a groin flap. **(C, D)** X-rays of the hand and foot. **(E–H)** Free metatarsal phalangeal joint. **(I)** The metatarsal bone and the proximal end of the first metacarpal were fixed by a plate, and the phalange of the heterotopic replanted thumb was fixed by a Kirschner wire. **(J–L)** The color of the thumb and flap indicates good blood supply.

The heterotopic replanted thumb was successfully replanted back to its original position 5 months after the injury, and the metatarsophalangeal joint of the second toe was harvested to reconstruct the first carpometacarpal joint(Figures 2A–H). The skin was cut, and the dorsal metacarpal superficial vein was found and connected to the vein of the heterotopic thumb, which was free for approximately 5 cm then was cut off, and the end was marked. The radial-side digital artery of the middle finger that anastomosed with the thumb artery was also freed and labeled. Debridement was done under the microscope to find the bilateral artery, the digital nerve, and the tendons. The second metatarsophalangeal joint was harvested from the left foot with a 3 cm × 1.5 cm flap, carrying the flexor and extensor tendons, the dorsal metatarsal artery, the dorsal veins, and the fibular nerve of the second toe (Figure 2I). The metatarsophalangeal joint was placed in the appropriate position, the metatarsal bone and the proximal end of the first metacarpal were fixed by a plate, and the phalange of the heterotopic replanted thumb was fixed by a Kirschner wire. The flexor and extensor tendons were repaired. The dorsal metatarsal artery, the radial side artery of the middle finger, and the radial artery were anastomosed under a microscope, and the dorsal vein of the thumb, the dorsal metacarpal superficial vein, and the cephalic vein were also anastomosed. The bilateral nerve of the thumb, the fibular nerve of the second toe, and the superficial branch of the radial nerve were repaired (Figures 2J–L). The wound was treated with postoperative plaster external fixation for 1 month.

### 2.3 Postoperative treatment and follow-up

After the severed finger was temporarily heterotopic replanted, the patient was sent to the microsurgery care unit and received symptomatic treatment. Postoperatively, heparin sodium (12,500 iu) was given once a day, poppycock (1 mL) was given every 8 h, and 500 mL of dextrose amino acid and other vascular antispasmodic and anticoagulant therapies were administered. Preoperatively and postoperatively, cephalosporin antibiotics were used to prevent infection for approximately 7 days. At the same time, postoperative rehydration and routine microsurgical care with other medication, such as hepcidium saponin and painkillers, were provided. In the first week, the dressing was changed 1–2 times a day with clean alcohol.

Close attention was paid to the coagulation activated partial thromboplastin time (aPTT) index, and according to the formula, body weight, the patient’s aPTT is maintained at a level slightly higher than normal. The doses of heparin sodium and other drugs were adjusted as needed. The patient was required to stay strictly in bed, elevate the affected limb, and strengthen nutrition. After 6 days of observation, the replanted finger showed a tendency to be stable, and the blood flow was good. The use of heparin sodium and other drugs was stopped. The frequency of dressing change with alcohol was changed to 1–2 days. The patient was then transferred to the general ward for treatment.

The patient began functional rehabilitation exercise 1 month after surgery. Rehabilitation included finger flexion and extension activities, abduction and adduction activities, finger fine activity training, such as palm function, pinch, grip, and other exercises, and gradual training according to the recovery situation. Follow-up and hand function assessment was conducted at 1 month, 3 months, 6 months, and 12 months postoperatively.

#### 2.3.1 Appearance

**FIGURE 3:**
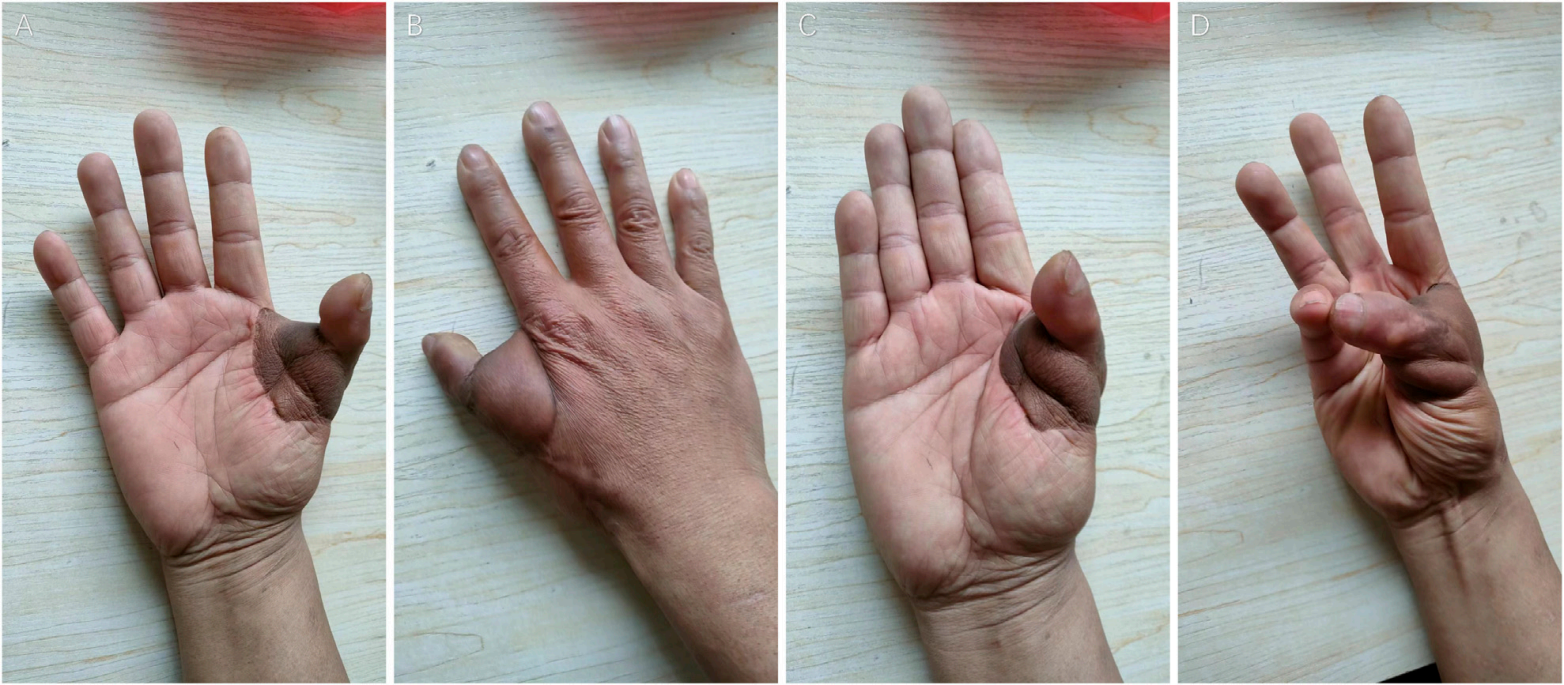
Ten-year follow-up of thumb heterotopic replantation: **(A)** Right thumb extension palmar view, **(B)** dorsal view, **(C)** right thumb flexion palmar view, and **(D)** thumb opposition function.

The heterotopic replanted thumb is slightly shorter than the normal thumb of the left hand. There is partial atrophy of the finger pulp. The nail grows well, the flap is soft and elastic, and the blood supply of the thumb and flap is good (Figures 3A, B).

#### 2.3.2 Range of motion

The reconstructed metacarpophalangeal joint function recovered well, and the abduction and adduction function was restored. The adduction angle was reasonable, and the range of motion was approximately 60–65°. However, the interphalangeal joint activity was poor in terms of the Chinese Association of Hand Surgery upper limb function evaluation criteria. The functional recovery score was recorded as metacarpophalangeal joint and interphalangeal joint range of motion (ROM) less than 90°, and the autonomic range of motion score of thumb was five points (Table 1, Figures 3C, D). The thumb can do pinching, grasping, and other fine movements. The patient could conduct basic activities of daily life, such as pinching needles, writing, and holding a cup, and scored 15 points (Table 1).

**TABLE 1:** Trial standards for evaluating upper limb partial function of the Hand Surgery Society of Chinese Medical Association.

Functional evaluation trial standard		Heterotopic replantation	Primary thumb replantation
1. Motor function	①Thumb opposition	10	10
	②Voluntary range of motion of the thumb joint	5	5
2. Activities of daily living: ADL		16	15
3. Sensory recovery		16	16
4. Circulatory state		10	10
5.Appearance		16	16
6. Return to work		7	10
Four scores, grade score Fine, 100–80; good, 79–60; poor, 59–40; inferior ˂ 40		80	82

#### 2.3.3 Sensory function restored

The sensory function of the heterotopic replanted thumb was mostly restored. The two-point discrimination was approximately 8 mm.

### 2.4 Comparison

**FIGURE 4:**
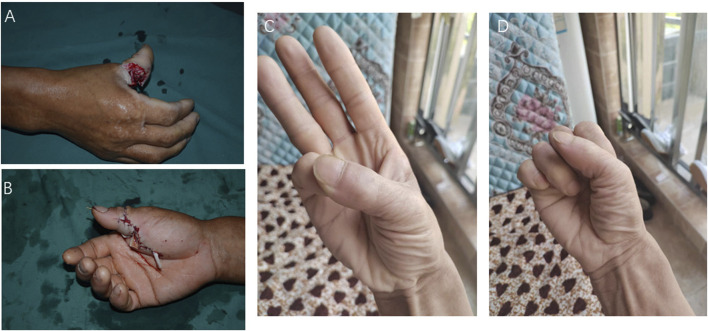
Patient with severed thumb: **(A, B)** Preoperative data and **(C, D)** photographs after thumb replantation.

The functional recovery of the heterotopic replanted thumb was compared to an emergency replantation done in the same period. A successful thumb replantation case with the same basic conditions and injury position as the heterotopic replantation case was selected for functional analysis. The patient was a male individual, 44 years old, and the right thumb was completely detached from the proximal phalanx with only approximately 1.5 cm dorsal skin connected (Figures 4A, B). The patient received emergency finger replantation, and the finger survived without further complications. Conventional treatment and functional exercise were taken like the heterotopic replanted thumb.

The thumb function was evaluated after 10 years of follow-up. The appearance of the thumbs was good, and a slight scar could be seen at the injury site. The range of motion is approximately 65–70°, but the interphalangeal joint movement was poor. The pinching and gripping of the thumb were good, and the sensory function was recovered. The two-point discrimination was approximately 8 mm (Figures 4C, D).

## 3 Discussion

With the development of microsurgery, the survival rate of finger and limb replantation has significantly improved, but finger replantation remains a daily challenge for hand surgeons. Although four cases of severed finger cryopreservation and replantation have been successfully applied in clinical practice, the cryopreservation effect of large composite tissue is poor, and the clinical application is limited (Wang et al., 2020; Wang Z. et al., 2019). Heterotopic replantation is another innovative way to save the severed finger in special circumstances (Bakhach et al., 2008). Temporary heterotopic replantation was first reported by Godina in 1986. It was applied to patients with complex injuries and those in critical condition who cannot undergo emergency replantation surgery. This method can preserve the necessary length of the patient’s limb in the first stage, improve the functional and esthetic effects of replantation in the second stage, and maximize the reconstruction of severed limbs (Cho and Higgins, 2019; Wang et al., 2006).

There have been numerous reports of rescuing amputated body parts by this method, including fingers, hands, forearms, feet, penises, testicles, scalp, etc. (Tu et al., 2018). However, a few articles describe the long-term functional recovery of heterotopic replanted hands and fingers. In a systematic review of ectopic foster-replantation, most patients experienced sensory recovery at the tip of the reconstructed limb at different follow-up times (Tu et al., 2018). However, limb function may not be fully restored in patients. According to case reports, finger and hand function recovery was poor in patients with ectopic foster-replantation (Cooper, 1997). Li reported the function of 2 heterotopic replantation fingers and the reconstructed thumb had good sensory recovery and good extension and flexion function, Explained the feasibility and important value of heterotopic replantation thumb (Li et al., 2008). There is no uniform consensus on indications of temporary heterotopic replantation.

Wang suggested that the contralateral limb is an acceptable site for temporary heterotopic replantation (Wang et al., 2006). Compared to other areas of foster care, such as the forearm and lower limbs, Tomlinson successfully used the radial blood vessels of the opposite forearm to foster three fingers on the affected side and reconstruct a hand without metacarpal bones after the replantation (Tomlinson et al., 2007). In our cases, the severed finger was placed on the dorsal side of the opposite healthy hand. The veins converged to three thick veins on the dorsal side of the hand, and the artery was anastomosed with the radial side digital artery of the middle finger as the diameter of the blood vessels is appropriate. In the second stage of replantation, the second metatarsophalangeal joint flap with tendons, blood vessels, and nerves was used for heterotopic thumb bridging replantation, which resulted in significant improvements in both aesthetics and function compared to patients who underwent direct replantation. Davide conducted a long-term follow-up analysis on the functional recovery of first-stage thumb replantation patients. More than 90% of patients returned to work, with different functional recoveries ranging from over 70% (Giardi et al., 2020; Unglaub et al., 2006). Thumb reconstruction is another possible way to save the thumb function (Adani and Woo, 2017). In our study, the appearance and function of the first-stage finger replantation and heterotopic replantation were compared during the 10-year follow-up. The patients with heterotopic replantation may face the possibility of multiple repair surgeries, while the patients with emergency replantation can gradually enter the recovery phase with fewer complications.

Thumb heterotopic replantation combined with free joint transplantation is another approach to preserving finger function. However, a successful surgery is merely the first step. Postoperative care and the prevention of risk factors are equally crucial. Postoperative complications, such as minor necrosis and superficial infection, were common during the implantation or replantation stage. Literature reports indicate there is a risk of vascular thrombosis after surgery for ectopic reimplantation, which can lead to finger ischemia (Tu et al., 2018). Additionally, anastomotic rupture, the possibility of acute ischemia, and recurrence of infection may occur, potentially resulting in surgical failure. The prevention of these risks has been described in the previous article. It should begin with close monitoring of the aPTT index after surgery. Administering anticoagulant, antispasmodic, and anti-infectious drugs, as well as ensuring strict bed rest, elevating the affected limb, changing medications, strengthening fluid intake and nutrition during the first week, and so on are important to the long-term success of the surgery.

The results of thumb heterotopic replantation combined with free joint transplantation show that this method is feasible. Because the flap at the metacarpophalangeal joint of the palm thumb was bloated, and there was partial atrophy of the finger pulp, the appearance of the heterotropic replanted thumb was worse than a normally replanted thumb. The length was similar to the normal thumb. The metacarpal and phalangeal joint activity, pinch, grip, thumb opposition function, and fingertip sensation were satisfactory.

Heterotopic thumb replantation mainly faces the problem of multiple surgeries, the economic burden, and the psychological pressure on patients. The method is suitable for patients with relatively intact fingers who are unable to undergo one-stage emergency replantation (Ercin et al., 2022).

## 4 Conclusion

Thumb heterotopic replantation combined with free joint transplantation is a feasible method for patients with severe hand injuries who are unable to undergo one-stage replantation. It is applicable to patients with devastating segmental or soft tissue injuries, difficulty in early assessment of the necrotic area of the injured limb for immediate revascularization, multiple organ injuries or hemodynamic instability that prevent immediate prolonged replantation, and heavy contamination of the amputation stump. Compared with other surgeries, such as finger reconstruction, it reduces relatively large trauma and is capable of preserving the function of the patient’s finger. Nevertheless, due to the currently small number of cases, it is impossible to demonstrate further effectiveness. We believe that the analysis of this study will provide new ideas and inspiration for the treatment of such patients in the future.

## Data Availability

The original contributions presented in the study are included in the article/Supplementary Material; further inquiries can be directed to the corresponding author.
