# Supplementary material for: Long-term follow-up of thumb reconstruction with a heterotopic replanted finger: case report and literature review
Source: Front Bioeng Biotechnol. 2024 Nov 8;12:1465108. doi: 10.3389/fbioe.2024.1465108 (PMC11581888; doi:10.3389/fbioe.2024.1465108)
Supplement: Supplementary file 1 [file Table1.DOCX]

| Functional evaluation trial standard | | heterotopic replantation | Primary thumb replantation |
| --- | --- | --- | --- |
| 1.Motor  function | ①Thumb opposition | 10 | 10 |
|  | ②Voluntary range of motion of thumb joint | 5 | 5 |
| 2 Activities of daily living: ADL | | 16 | 15 |
| 3.Sensory recovery | | 16 | 16 |
| 4.Circulatory state | | 10 | 10 |
| 5.Appearance | | 16 | 16 |
| 6.Return to work | | 7 | 10 |
| Four scores, grade score:  Fine,100～80 score good,79～60 score Poor,59～40 score Inferior,＜40 score | | 80 | 82 |
